# *Lacticaseibacillus rhamnosus* CA15 (DSM 33960) strain as a new driver in restoring the normal vaginal microbiota: A randomized, double-blind, placebo-controlled clinical trial

**DOI:** 10.3389/fsurg.2022.1075612

**Published:** 2023-01-09

**Authors:** Agnese Maria Chiara Rapisarda, Alessandra Pino, Raffaela Luisa Grimaldi, Cinzia Caggia, Cinzia Lucia Randazzo, Antonio Cianci

**Affiliations:** ^1^ Department of General Surgery and Medical Surgical Specialties, University of Catania, Catania, Italy; ^2^ ProBioEtna SRL, Spin off of the University of Catania, Catania, Italy; ^3^ Department of Agricultural, Food and Environment, University of Catania, Catania, Italy; ^4^ CERNUT, Interdepartmental Research Centre in Nutraceuticals and Health Products, University of Catania, Catania, Italy

**Keywords:** vaginal microbiota, quality of life, dysbiosis, probiotics, women's health, lactobacilli

## Abstract

Probiotics play a key role in maintaining the health of the female reproductive tract, representing a promising alternative to safeguard or restore the homeostasis of the vaginal microbiota. The present randomized double-blind placebo-controlled study was performed to evaluate the ability of the potential probiotic *Lacticaseibacillus rhamnosus* CA15 (DSM 33960) strain, orally administrated, to balance the vaginal microbiota of women with vaginal dysbiosis. Sixty women, with signs and symptoms of vaginal dysbiosis, were recruited and randomly allocated to receive oral capsules containing the *L. rhamnosus* CA15 (DSM 33960) strain at 1.0 × 10^10^ colony-forming units or placebo once daily for 10 days. Clinical and microbiological parameters were evaluated in three scheduled appointments: at baseline (T0), 10 days after the start of the treatment (T1), and 30 days after the end of the treatment (T2). In addition, the quality of life, through the WHO quality of life assessment questionnaire, was assessed at baseline (T0) and 30 days after the end of the treatment (T2). The probiotic was well tolerated and no side effects were reported. The oral consumption of the potential probiotic *L. rhamnosus* CA15 (DSM 33960) strain determined a significant improvement of both clinical signs and symptoms not only 10 days after the start of the treatment (T1) but also 30 days after the end of the treatment (T2). A significant reduction of potential pathogens and a concomitant increase of lactobacilli was revealed, by microbial count, at both T1 and T2 sampling times. In addition, the enhancement of the perceived physical health, social relations, and environment was reported. Differently, in placebo group clinical and microbiological parameters as well as quality of life remained almost unchanged. The potential probiotic *L. rhamnosus* CA15 (DSM 33960) strain could be a safe and effective approach to restore and maintain a balanced vaginal microbiota.

## Introduction

The vaginal microbiota (VM) is a dynamic ecosystem consisting of bacteria, viruses, archaea, fungi, and protozoa (1, 2). Under physiological conditions, the VM plays a pivotal role in women's health acting, as a frontline defender against pathogenic microorganisms. It is widely accepted that, in healthy women of reproductive age, a balanced VM is dominated by lactobacilli (3).

Lactobacilli, by producing metabolites (e.g., lactic acid), antimicrobial substances, such as bacteriocins and hydrogen peroxide, and interacting with the host innate immune system, can protect the vaginal mucosa from non-indigenous and potentially harmful microorganisms (4). In the case of non-*Lactobacillus*-dominant VM, a high risk for adverse health outcomes is reported. In particular, the reduction or loss of lactobacilli is associated with vaginal infections, such as bacterial vaginosis (BV), vulvovaginal candidiasis (VVC), mixed vaginitis, sexually transmitted infection (STI) and spontaneous preterm birth (5–7).

BV is associated with an increased abundance of facultative and anaerobic microorganisms which are responsible for increased vaginal discharge, impaired vaginal pH and a characteristic Foul-smelling “fishy” odor (8). The prevalence of BV is between 23% and 29% of women of reproductive age (9, 10).

In VVC vulvar pruritus and burning as well as vaginal dyspareunia and dysuria are the main clinical symptoms. It is estimated that more than 75% of women experienced at least one episodes of VVC in their lifetime and that recurrences are very common (11–13). Standard-of-care treatment for BV includes the use of metronidazole (topical or oral) or clindamycin (topical) (14) whereas polyenes (e.g., nystatin) and azoles are usually administrated in case of VVC (15). Unfortunately, antibiotics and antifungal drugs are not always effective since microorganisms responsible for BV and VVC can resist by forming biofilms or by acquiring resistance (16, 17). In addition, antibiotics, not selectively acting against pathogens, can negatively affect endogenous lactobacilli and in turn potentially promote BV and VVC recurrence (18). Based on these evidence, alternative strategies, to balance the VM and maintain a healthy vaginal ecosystem, are needed. Data emerging from clinical trials and meta-analyses have suggested that probiotics may play a positive role in the treatment vaginal dysbiosis (19–22). However, it should be emphasized that the various probiotic strains have different *in vitro* properties and to be effective, not only a certain strain must reach and colonize the human vagina but should have specific antimicrobial properties. In addition, to determine whether subjects are responders or non-responders to a specific probiotic, attention must be paid to their effectiveness in the resolution of vaginal symptoms and their impact on the quality of life.

The present randomized double-blind placebo-controlled study was performed to assess the efficacy of the potential probiotic *L. rhamnosus* CA15 (DSM 33960) strain, orally administrated, to treat vaginal dysbiosis. According to that, clinical parameters and women's subjective vaginal symptoms were evaluated. In addition, the effect on general health was evaluated by the WHOQOL-BREF questionnaire.

## Materials and methods

### Study design and population

A randomized double-blind placebo-controlled trial was performed at the Department of General Surgery and Medical Surgical Specialties, University of Catania (Italy). The study was conducted according to the Good Clinical Practices and the World Medical Association (WMA) policy regarding the Ethical Principles for Medical Research Involving Human Subjects, as stated in the Declaration of Helsinki. The study protocol was approved by the Institutional Review Board (IRB) of the University hospital and by local Ethical Committee (registration number 113/2022/PO).

Healthy women of reproductive ages (18–45 ages) with vaginal signs and symptoms of vaginal infections, confirmed by clinical and microbiological analyses, were enrolled according to the inclusion and exclusion criteria detailed in Table 1.

**Table 1:** Description of inclusion and exclusion criteria.

Inclusion Criteria	Exclusion Criteria
Fertile age (18–45 years).	Presence of sexually transmitted disease due to *Chlamydia*, *Neisseria gonorrhoeae* or *Trichomonas vaginalis*.
Presence of at least one vaginal symptom: (leucorrhoea, burning, itching, erythema/edema or subjective vaginal discomfort).	Presence of specific vaginitis related to acute candidiasis.
Clinical evidence of vaginal dysbiosis: [at least 3 Amsel criteria or Nugent score ≥7 or lactobacillary grade (LBG) ≥ 2].	Clinically apparent herpes simplex infection.
Signed informed consent.	Precancerous lesions due to Human papillomavirus.
Not participating in other clinical studies.	Human immunodeficiency virus infection
Willingness to take the investigational product or placebo.	Confirmed diagnosis of pelvic inflammatory disease (PID).
Willingness to collaborate in completing the study procedures.	Recent use of antibiotic, antifungal drugs (less than one month)
** **	Recent consumption of probiotics or food containing probiotics.
** **	Recent use immunosuppressive drugs (less than one month).
** **	Actual or recent use of vaginal contraceptives.
** **	Pregnancy or breastfeeding.
** **	Use of douching.
** **	Hypersensitivity or allergy to any ingredient of investigational product or placebo.
** **	Chronic diseases.
** **	Neoplastic disease.
** **	Diabetes
** **	Genital tract bleeding.

**Figure 1:**
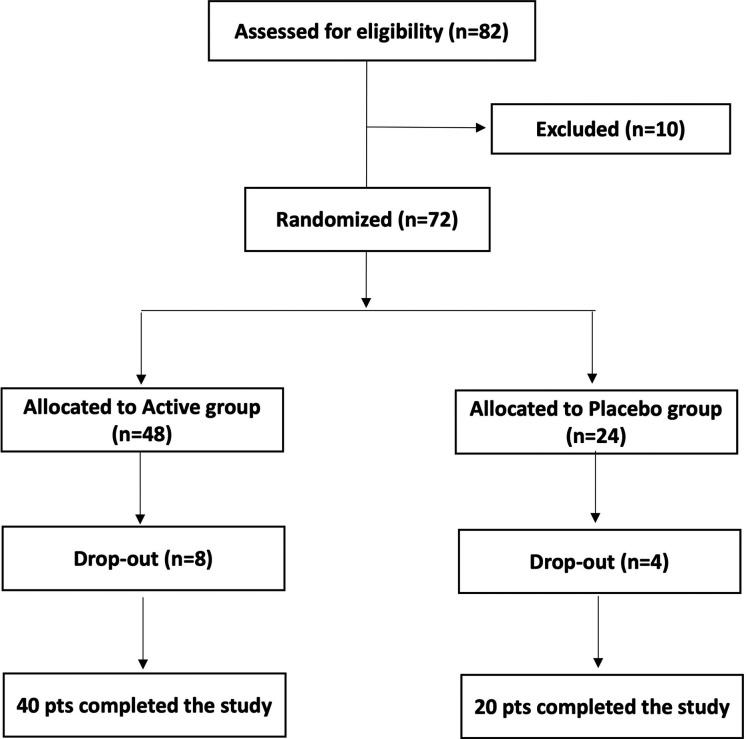
Study flow chart.

Women were informed about the study protocol, procedures, investigational product and potential risks of treatment as well as about the opportunity to freely leave the study at any time. Each woman participating in the study signed an informed consent form for data collection. Personal data were anonymously treated following Italian law guaranteeing privacy. The participation in the study was closely voluntary and no remuneration was offered. Figure 1 shows the study flow chart. All enrolled women were randomly divided into 2 groups, Active and Placebo, according to a computer-randomized scheme (2:1 ratio). Patients allocated to the Active group took 1 oral capsule for 10 consecutive days. Each active capsule contained 10 log cfu/g of the *L. rhamnosus* CA15 (DSM 33960) probiotic strain. Placebo consisted of an identical capsule containing corn starch as excipient.

Examinations of each woman were scheduled in three appointments: baseline (T0), 10 days after the start of the treatment (T1), and 30 days after the end of the treatment (washout, T2). In addition, the quality of life was assessed through the WHO quality of life assessment questionnaire (23) at baseline (T0) and 30 days after the end of treatment (T2). Forty (*n* = 40) patients allocated to Active group and twenty (*n* = 20) patients allocated to Placebo group completed the study.

During the whole study, each woman was requested to accurately record in a personal diary any potential adverse reaction or any use of medication. Patients were also warned to promptly report any worsening of symptomatology. In cases of significant discomfort and clinical or self-reported worsening of symptoms, women were immediately excluded from the experimental observation, and they were treated with conventional therapies.

### Clinical evaluation

Demographic data about each woman, as information about age, smoking habit, relationship status, body mass index, childbirth history, sexual activity, contraceptive use, disease and allergy histories, were collected at the time of consent.

Clinical signs and symptoms, were evaluated through a severity score on a scale of 0 (absent or normal) to 3 (severe), and vaginal fluid pH, measured using pH test strips (McKesson, San Francisco, CA, USA) were recorded at T0, T1, and T2 sampling times.

The Nugent score was assessed on a 10-point scale, performing a Gram stain followed by optical microscopic observation under oil immersion.

The presence of at least three of the Amsel criteria was assumed for BV. A Nugent score of 0–3 was interpreted as *Lactobacillus*-predominant normal vaginal microbiota, a score of 4–6 was considered as intermediate, and a score of 7–10 was assumed as BV-like condition (24). Lactobacillary grade (LBG) was evaluated according to Donders classification: LBG I was assumed for a normal microbiota with predominant lactobacillary morphotypes, LBG II corresponded to a reduction of the lactobacillary population and a concomitant presence of other bacteria, whereas LBG III was defined as an abnormal flora which consists of numerous other bacteria with the absence of lactobacillary population (25).

The WHOQOL-BREF questionnaire was used to assess the quality of life (QoL). It is a patient-reported instrument that can evaluate the global health status of patients independently of disease across 4 health domains (Physical, Psychological, Social relations, and Environment). Overall, it includes 26 questions considered as the most important among the 100 questions present in the predecessor's. The patient's recall period covers the past 2 weeks. The first two questions of the WHOQOL-BREF do not correspond to a domain but provide a global assessment of the quality of life. High scores in each of the domains correspond to greater perceived quality of life.

All data were recorded in an appropriate database form, including different sections related to personal data, patient's medical history, and information about intake of any concomitant drugs, symptoms related to vaginal dysbiosis, Amsel criteria, Nugent score, and microbiological count.

For results interpretation, the main endpoints for resolution of the pathological condition were defined as the absence of vaginal symptoms, negative results for at least 2 Amsel criteria, Nugent score less than 4, a significant reduction of potentially pathogenic bacteria and an increase of lactobacilli.

### Vaginal discharge samples collection

Vaginal discharge samples were obtained from the lateral vaginal wall and the posterior fornix using sterile cotton-tipped swabs. For each participant, a total of three swabs were collected at T0, T1, and T2 sampling times in order to perform the microscopic examination of the fresh smear (detection of clue cells and Gram staining), the evaluation of the Nugent score and whiff-amine test, using two different glass slides, another swab, and the microbiological count as reported below. Vaginal samples were collected at the Department of General Surgery and Medical Surgical Specialties, Gynecological Clinic, University of Catania (Catania, Italy), and immediately transferred, under refrigerated conditions, to the Laboratory of ProBioEtna Srl (Catania, Italy).

### Microbiological analysis of vaginal bacterial biota

At each sampling time (T0, T1, and T2), vaginal discharge samples were collected, using swabs filled with the transport medium Transystem Amies Clear (Biolife, Milan, Italy), and were subjected to culture-dependent analysis according to Pino and co-workers (26, 27). In detail, after dislodging bacterial cells in sterile phosphate-buffered saline (PBS), 10-fold dilutions were made and plated using the following agar media and conditions: Rogosa SL agar (Biolife, Milan, Italy) for *Lactobacillus* counts, incubated at 35–37 °C for 40–48 h; Streptococcus Selective Agar (Biolife, Milan, Italy) for the enumeration of streptococci, incubated at 32 °C for 24 h; Columbia Blood Agar base (Oxoid, Milan, Italy), supplemented with Gardnerella Vaginalis Selective Supplement (Oxoid, Milan, Italy), incubated at 37 °C for 40–48 h for *Gadnerella* spp. count; MacConkey Agar Mug (Biolife, Milan, Italy) incubated at 37 °C for 16–18 h for *Escherichia coli*; Mannitol Salt Agar (Oxoid, Milan, Italy) incubated at 32 °C for 48 h for the count of staphylococci; Slanetz Bartley Agar (Biolife, Milan, Italy) incubated at 37 °C for 48 h for enterococci; and Chromogenic Candida Agar (Biolife, Milan, Italy), incubated at 35–37 °C for 18–48 h, for the count of *Candida* spp.

Microbiological count was performed in triplicate and results were reported as mean log cfu/ml and standard deviation.

### Statistical analysis

Wilcoxon, Mann–Whitney and McNemar tests were performed through the SciPy Python library (Python 3.9.12, scipy 1.8.0) to detect significant differences among clinical parameters. The Nugent score was coded as an ordinal variable assigning 0 to the 0–3 score, 1 to the 4–6 score, and 2 to the 7–10 score.

Time was treated as an ordinal variable. Nugent score and time were correlated using Kendall's test (Kendall's Tau-b, from SciPy). Distribution of the sample means was obtained through bootstrap and represented *via* violin plots. Clinical and microbiological characteristics were compared using the Student's *t*-test, Fisher's exact test and Chi-squared test when appropriate. One-way ANOVA followed by Tukey's test, performed using SPSS Version 25.0 (Armonk, NY: IBM Corp.), was applied to detect differences among mean values of the detected microbial groups. Differences were considered statistically significant at *p*-value <0.05.

## Results

### Demographic and clinical baseline data

After the preliminary evaluation, 82 women were selected to be eligible satisfying the inclusion criteria reported in Table 1. However, after completing the clinical and microbiological baseline assessment, 10 subjects were excluded for unconfirmed microbiological diagnosis of vaginal dysbiosis. Of the 72 participants, 60 completed the study by adhering to the therapeutic regime and undergoing scheduled follow-up visits. The remaining 12 cases (8 active and 4 placebo) left the study for the following reasons: lost to follow-up, fever, seasonal flu, pregnancy, lack of symptoms control.

No severe adverse events were recorded for the entire observational period and nobody, among the participants, was excluded from the study due to the onset of adverse events.

Overall, 60 women, aged from 19 to 45 years (mean age of 32.93 ± 7.58 years), completed the study and were therefore evaluated for efficacy analysis.

Each woman had regular menstrual cycles (minimum, 21 days; maximum, 35 days), the majority were sexually active (77.66%) and were included in the ideal healthy bodyweight range (Mean BMI = 23.73 ± 3.10). Seventy-seven (77) percentage of women were contraceptive users. The demographic characteristics in terms of age, body mass index (BMI), sexual activity, smoking, and contraceptives use, as well as the clinical findings resulting from the analysis of the study population, are shown in Tables 2, 3.

**Table 2:** Baseline demographic characteristics of participating women (*n* = 60).

			Total sample (*n* = 60)	Active (*n* = 40)	Placebo (*n* = 20)	*p* value
**Demographic characteristics**						
**Age**			32.93 3 ± 7,58	32.85 3 ± 7,55	33.10 3 ± 7,85	0.91
**Sexual activity**			46 (76.66%)	31 (77.50%)	15 (75.00%)	1.00
**Smoking**			18 (22.50%)	13 (32.25%)	5 (25.00%)	0.77
**Body mass index (kg/m2)**			23.73 ± 3.10	23.33 ± 3.46	22.53 ± 1.74	0.11
		**<18.5**	1 (1.67%)	1 (2.50%)	0 (0.00%)	1.00
		**18.5–24.9**	38 (63.33%)	23 (57.50%)	15 (75.00%)	0.25
		**25–29.9**	19 (31.67%)	14 (35.00%)	5 (25.00%)	0.56
		**≥30**	2 (3.33%)	2 (5.00%)	0 (0.00%)	1.00
**Contraceptive use**			46 (76.66%)	29 (72.50%)	17 (85.00%)	0,35
	**Oral**		13 (21.66%)	11 (27.50%)	2 (10.00%)	0.19
	**Barrier**		19 (31.66%)	11 (27.50%)	8 (40.00%)	0.38
	**Others**		14 (23.33%)	7 (17.50%)	7 (35.00%)	0,20

Data are presented as means ± SD and percentages.

**Table 3:** Baseline clinical and microbiological characteristics of the study sample (*n* = 60).

		Total sample (*n* = 60)	Active (*n* = 40)	Placebo (*n* = 20)	*p* value
**Clinical and microbiological characteristics**					
Vulvovaginal signs and symptoms	Leucorrhoea	57 (95.00%)	37 (92.50%)	20 (100.00%)	0.54
	Burning	49 (81.67%)	32 (80.00%)	17 (85.00%)	0.73
	Itching	47 (78.33%)	31 (77.50%)	16 (80.00%)	1.00
	Vulvovaginal Erythema/Edema	43 (71.67%)	28 (70.00%)	15 (75.00%)	0.77
	Subjective vulvar discomfort	56 (93.33%)	36 (90.00%)	20 (100.00%)	0.29
Amsel Criteria	Homogenous vaginal discharge	54 (90.00%)	35 (87.50%)	19 (95.00%)	0.65
	Clue cell presence	42 (70.00%)	25 (62.50%)	17 (85.00%)	0.08
	Positive amine test	32 (53.33%)	14 (35.00%)	18 (90.00%)	<0.005
	Vaginal pH > 4.5	37 (61.67%)	20 (50.00%)	17 (85.00%)	0.01
Nugent score	0–3	0 (0.00%)	0 (0.00%)	0 (0.00%)	1.00
	4–6	1 (0,16%)	0 (0.00%)	1 (5.00%)	0.33
	7–10	59 (98.33%)	40 (100.00%)	19 (95.00%)	0.33
Lactobacillary grade	I	0 (0.00%)	0 (0.00%)	0 (0.00%)	1.00
	II	20 (33.33%)	12 (30.00%)	8 (40.00%)	0.56
	III	40 (66.67%)	28 (70.00%)	12 (60.00%)	0.56

Data are presented as means ± SD and percentages.

Baseline anamnestic and clinical data regarding the patients allocated to Active and Placebo groups were statistically compared. The two groups were homogeneous for age, parity and other clinical and instrumental analysed parameters allowing further analysis at follow-up (Table 2).

Concerning vulvovaginal signs and symptoms, leucorrhoea and vulvar discomfort were generally the most frequent, occurring, in the recruited population, with percentages of 95% and 93%, respectively; symptoms of burning, itching and vulvovaginal erythema were reported in 82%, 78% and 72% of cases, respectively. At baseline, no statistically significant differences were found between the two groups for the frequency of the evaluated symptoms.

Moreover, the percentage of patients who satisfied at least three Amsel criteria was 80%; among these, positive amine test and vaginal pH > 4.5 were the parameters showing statistically significant differences in frequency (*p*-value <0.005 and *p*-value = 0.01, respectively). Considering the clue cell presence, we found a similar trend (*p*-value = 0.08).

Almost all the enrolled patients (98%) showed Nugent score between 7 and 10.

Finally, none of the examined women showed Lactobacillary grade I. Almost 30% of women of both groups presented Lactobacillary grade II while the remaining had Lactobacillary grade III. No statistical differences were found among the active and placebo group for this parameter.

### Diagnostic parameters

**Figure 2:**
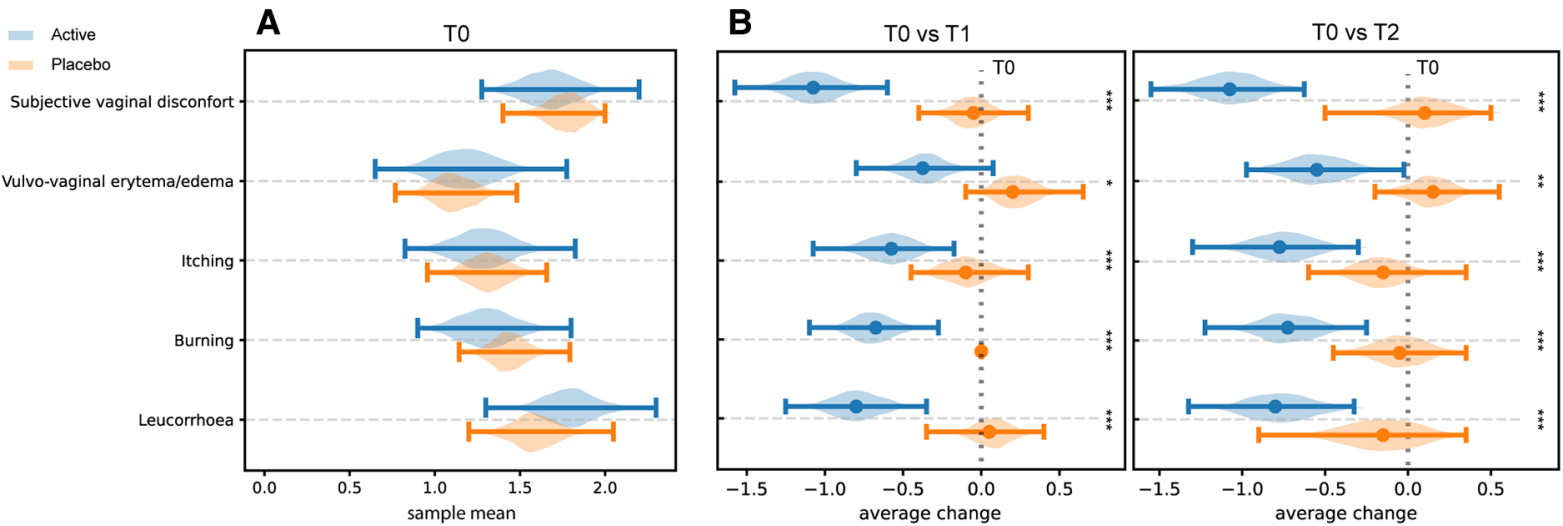
Perception of clinical symptoms. Distribution of the bootstrapped mean values related to the score obtained by the assessment of the intensity of clinical signs and symptoms at T0 in both groups (Panel **A**). Differences in the distribution of mean values T1 and T2 compared with T0 in the two different groups (Panel **B**). * = *p* ≤ 0.05, ** = *p* ≤ 0.01, *** = *p* ≤ 0.001.

Results from clinical signs and symptoms (leucorrhoea, burning, itching, vulvovaginal erythema/edema, and subjective vaginal discomfort) recorded at T0, T1, and T2 sampling times are shown in Figure 2 (panels A and B). At the baseline assessment, women allocated to the Placebo group showed lower mean values for vulvovaginal erythema/edema, itching and burning, while the assessment of the vaginal discomfort and leucorrhea exhibited similar scores by the two groups of women (Figure 2A).

**Figure 3:**
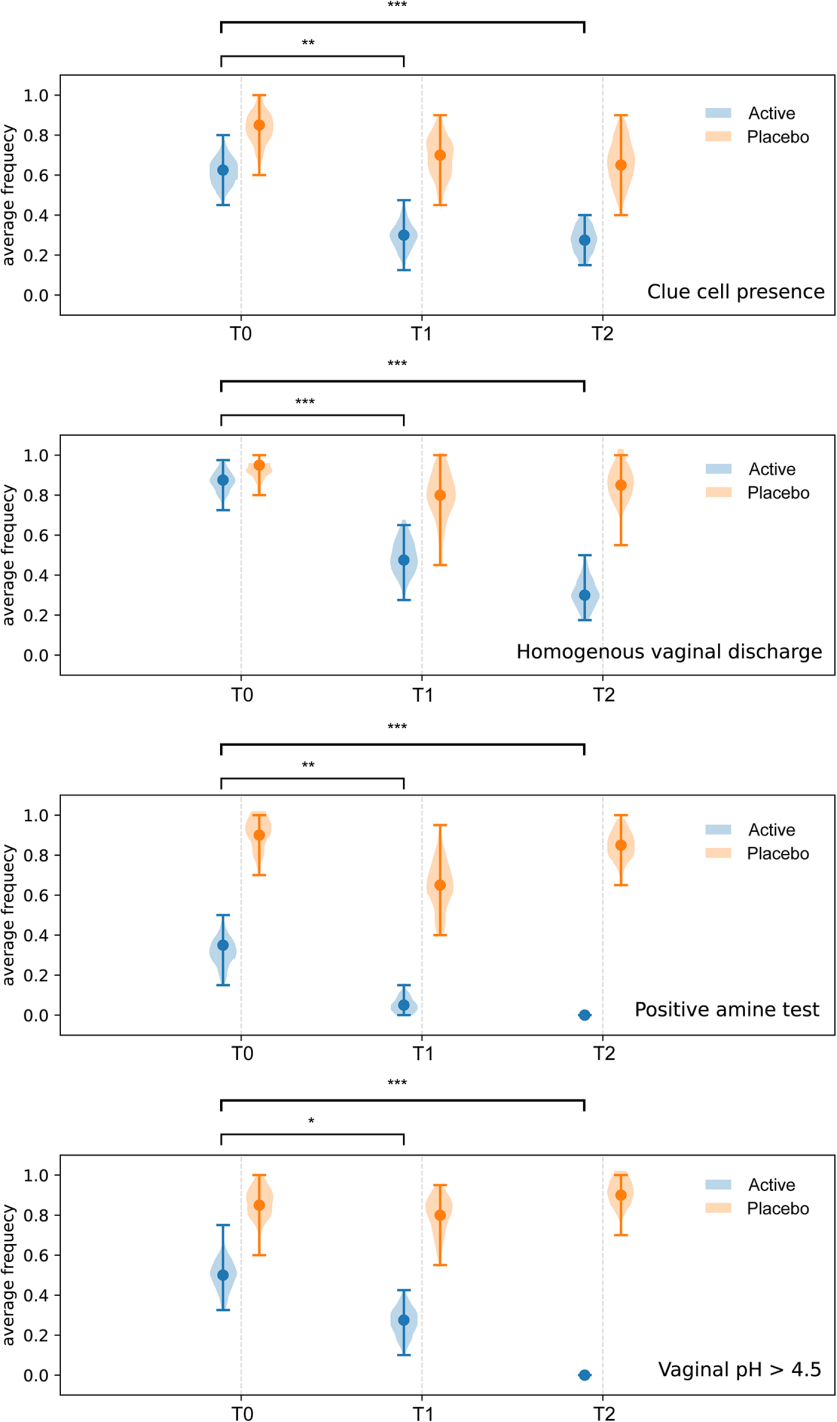
Amsel criteria. Comparison of Amsel criteria between treatment and placebo groups at baseline (T0), 10 days after the start of the treatment (T1) and 30 days after the end of the treatment (T2). * = *p* ≤ 0.05, ** = *p* ≤ 0.01, *** = *p* ≤ 0.001.

**Figure 4:**
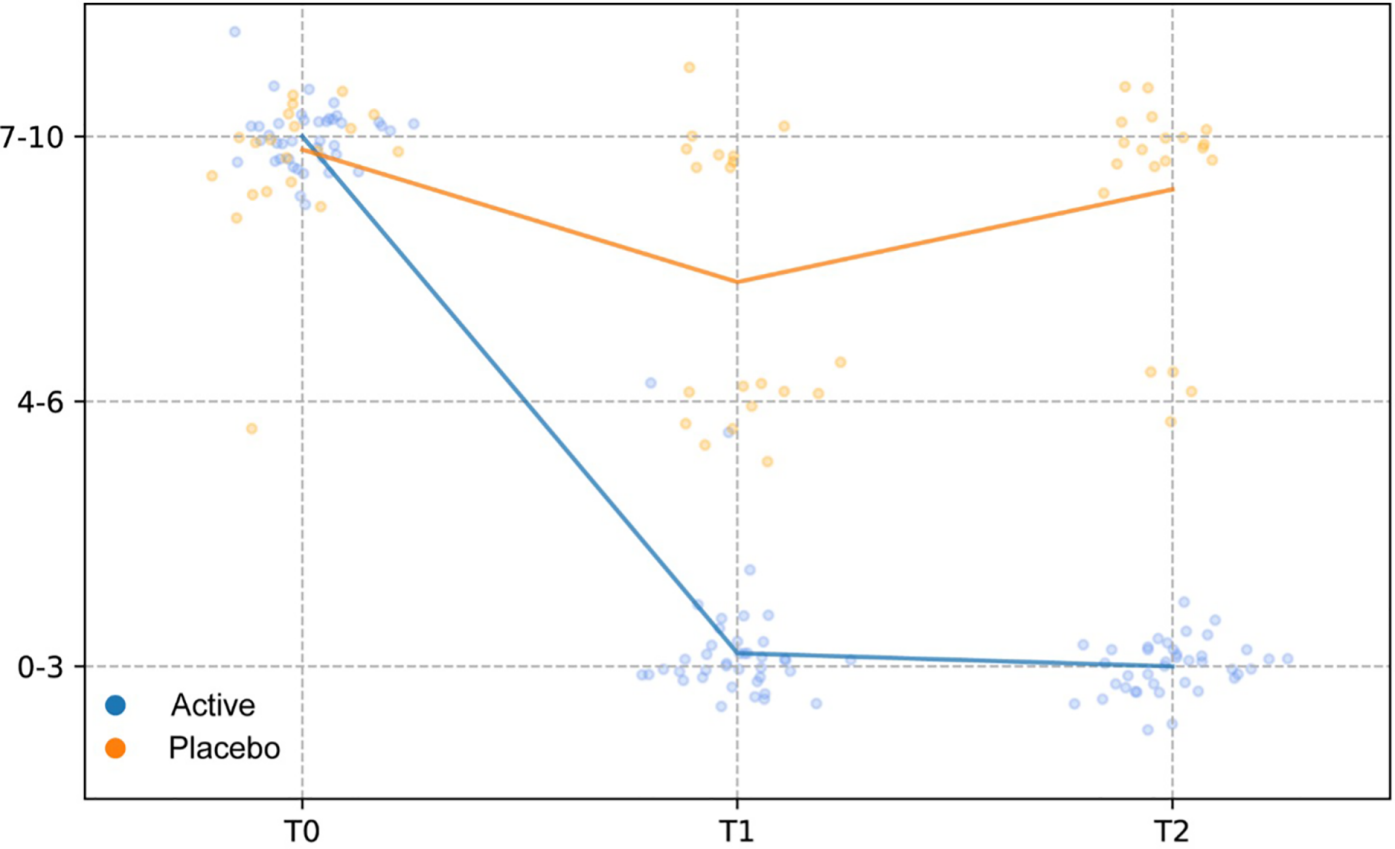
Nugent score. Comparison of Nugent scores between treatment and placebo groups at baseline (T0), 10 days after the start of the treatment (T1) and 30 days after the end of the treatment (T2). The Nugent scores were discretized and correlated by computing the Kendall rank correlation coefficient.

Compared to baseline (T0), a statistically significant reduction of the scores related to self-reported symptoms was reported in group Active 10 days after the start of the treatment (T0 vs. T1) (Burning *p*-value = 6.46 × 10^−5^; Itching *p*-value = 0.0012; leucorrhea = 4.56 × 10^−5^; Subjective vaginal discomfort *p* = 1.66 × 10^−6^; Vulvo-vaginal erythema/edema *p*-value = 0.0175). Substantially unchanged results were obtained 30 days after the stop of treatment (T0 vs. T2). On the other hand, scores registered in Placebo group, at both T1 and T2 sampling times, were much closer to those observed at T0 and not statistically significant (Figure 2B). As showed in Figure 3, all the Amsel criteria showed a decreasing trend in Active group at T1 sampling time compared to T0 (Clue cell presence *p*-value = 0.0023; Homogenous vaginal discharge *p*-value = 3.05 × 10^−5^; Positive amine test *p*-value = 0.0018; Vaginal pH > 4.5 *p*-value = 0.049). The aforementioned criteria decreased also at the T2 follow-up (Clue cell presence *p*-value = 0.0005; Homogenous vaginal discharge *p*-value = 2.38 × 10^−7^; Positive amine test *p*-value = 0.0001; Vaginal pH > 4.5 *p*-value = 1.91 × 10^−6^). Therefore, in only 2.7% of participants belonging to the Active group, at least three Amsel criteria were satisfied at T1% and 100% of women reported less than 3 Amsel criteria at the T2 follow-up. In Placebo group, all Amsel criteria remained almost unchanged compared to T0 (Figure 3). In addition, as displayed in Figure 4, a significant reduction in the Nugent score was detected at both T1 and T2 sampling times in Active group. More interesting, at T2 sampling time, all participants allocated to Active group showed Nugent score between 0 and 3 (Figure 4). Scores of this group at all times resulted to be negatively correlated (Kendall's *τ* = −0.817, *p*-value = 1.578 × 10^−21^) over time. Scores of Placebo group were not correlated since they underwent only a slight and not significant fluctuation at T1 (Figure 4).

**Figure 5:**
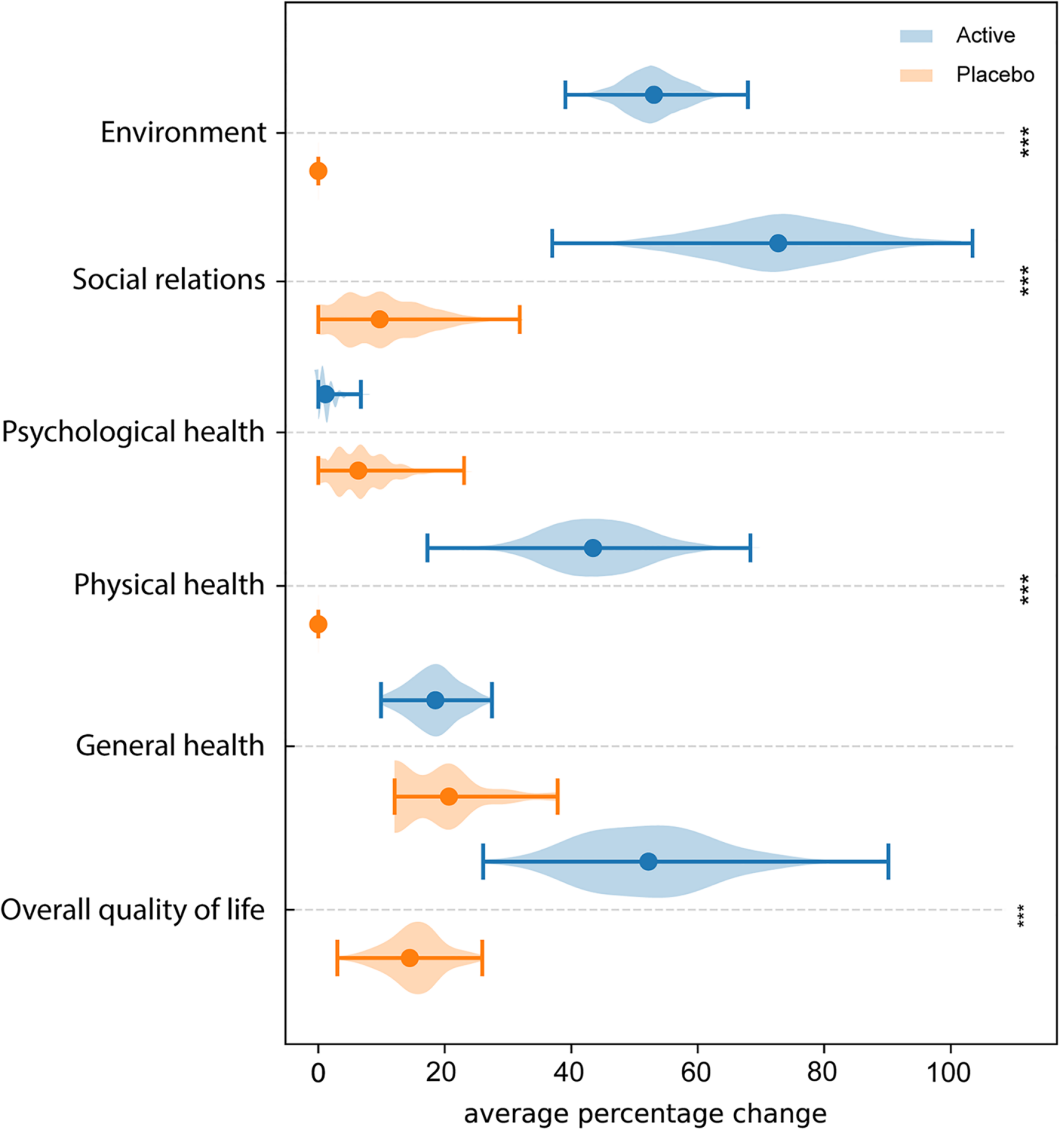
WHOQOL-BREF questionnaire. Average percentage difference between QoL domains assessed at T0 and T2. * = *p* ≤ 0.05, ** = *p* ≤ 0.01, *** = *p* ≤ 0.001.

Figure 5 shows the results of QoL among the two groups of women. The analysis of QoL domains showed, in Active group, a significant improvement in physical health (*p*-value = 3.42 × 10^−6^), social relations (*p*-value = 5.38 × 10^−6^), environment (*p*-value = 2.51 × 10^−8^) and overall QoL (*p*-value = 5.5 × 10^−5^). Differently, no significant variations were observed in Placebo group among the different domains.

### Bacterial biota composition by microbial count

The composition of the bacterial biota of enrolled patients, allocated to Active and Placebo groups, at baseline (T0), 10 days after the start of the treatment (T1), and 30 days after the stop of the treatment (T2) is reported in Table 4. Overall, at baseline (T0) all enrolled participants, allocated to both Active and Placebo groups, had an imbalanced microbiota dominated by potentially pathogenic bacteria with reduced cell density of lactobacilli. In the Active group, the oral administration of the *L. rhamnosus* CA15 (DSM 33960) strain for 10 days (T1), determined a statistically significant reduction of the investigated pathogens. On the contrary, a significant increase, of about 4 log units, of lactobacilli count was detected (Table 4). Thirty days after the end of the treatment (T2), the vaginal microbiota composition was quite stable and similar to those observed at T1 sampling time. In fact, comparing the data obtained at T1 and T2 sampling times, no significant differences were detected in the bacterial biota composition suggesting that the balanced condition, reached at T1, was not perturbed (Table 4). Different behaviour was detected in Placebo group. In fact, no statistically significant differences were detected in the bacterial biota composition (*p* > 0.05). High count of potentially pathogenic bacteria and low cell density of lactobacilli were detected during the whole study (Table 4).

**Table 4:** Bacterial biota composition and ANOVA significance of vaginal discharge samples collected from patients allocated in active and placebo group at baseline (T0), 10 days after the start of the treatment (T1), and 30 days after the stop of the treatment (T2).

Microbial groups	Active group						Placebo group					
	T0	T1	T2	p* T0 vs. T1	p* T1 vs. T2	p* T0 vs. T2	T0	T1	T2	p* T0 vs. T1	p* T1 vs. T2	p* T0 vs. T2
*Lactobacillus* spp.	3.75 ± 0.08	7.68 ± 0.16	7.81 ± 0.24	6.3 × 10^−6^	0.550835	2.23 × 10^−5^	4.04 ± 0.16	4.18 ± 0.18	3.92 ± 0.18	0.465382	0.223335	0.522061
*Enterococcus* spp.	5.18 ± 0.11	2.14 ± 0.15	2.21 ± 0.11	2.18 × 10^−5^	0.619159	1.15 × 10^−5^	5.04 ± 0.38	5.12 ± 0.41	5.34 ± 0.10	0.862471	0.498959	0.345368
*Staphylococcus* spp.	3.98 ± 0.22	1.80 ± 0.12	1.85 ± 0.17	0.000246	0.730142	0.000403	2.6 ± 0.32	2.36 ± 1.08	2.45 ± 0.15	0.775459	0.912751	0.573728
*Gardnerella* spp.	4.75 ± 0.21	1.98 ± 0.24	2.04 ± 0.10	0.000263	0.751912	8.4 ×10^−5^	4.15 ± 0.30	4.39 ± 0.31	4.3 ± 0.18	0.47532	0.736717	0.578468
*Candida* spp.	3.79 ± 0.17	1.28 ± 0.19	1.16 ± 0.13	0.00016	0.487949	6.46 ×10^−5^	3.93 ± 0.59	3.94 ± 0.44	4.09 ± 0.36	0.990367	0.716511	0.755271
*Escherichia coli*	4.12 ± 0.15	0.97 ± 0.13	1.08 ± 0.20	2.47 ×10^−5^	0.568684	6.54 ×10^−5^	3.22 ± 1.10	3.83 ± 0.52	3.77 ± 0.51	0.516909	0.912524	0.556048

Data are shown as mean log cfu/ml and standard deviation.

* Statistical significance *p* < 0.05.

## Discussion

In recent years there has been a growing interest towards the effectiveness of probiotics to boost women's health (28). Their use has gradually gained scientific acceptance, also in consideration of the widespread concerns linked to antibiotic resistance. Different probiotic formulations have been widely tested, for their potential effects on several gynaecological affections, including vaginal disease, endometriosis, miscarriage, preterm birth as well as gestational diabetes, and Polycystic ovary syndrome (PCOS) (26, 29–32).

Data emerging from preclinical research have supported that the supplementation of probiotic strains represents a strong rationale for the restoration of homeostasis in an unbalanced vaginal microbiota (33, 34). The actual mechanism of action of lactobacilli in the vagina is probably multifactorial, including immunomodulation, production of antimicrobial compounds, competition for nutrients and for the adhesion sites (35). Moreover, substantial experimental proof exists about vaginal colonization by lactobacilli following oral intake (36).

Nevertheless, data, emerging from previous prospective, randomized, double-blind, placebo-controlled trials supported clinical efficacy of probiotics in treatment of vaginal disease (37, 38). Only few studies have compared the efficacy of probiotics regimen alone vs. antibiotics in the treatment of vaginal dysbiosis (35, 37, 39).. Likewise, much less so far has been defined for the treatment of VVC and available studies have shown conflicting results and several limitations linked the suboptimal quality of design, absence of placebo group, high heterogeneity for probiotic strains, dose and treatment duration (18, 40).

Vaginal complaints are one of the foremost reasons for women seeking gynecological care (41). The evaluation has traditionally been oriented toward the detection of VVC, BV, and AV, according to specific clinical and microbiological criteria. But these three conditions do not account for all the clinical symptoms, treatment failures, and relapses.

It is important to highlight that vaginal dysbiosis is broad in nature, it comprises a panel of conditions that have, as their common denominator, the reduction of lactobacilli, and can assume the expression of BV, aerobic vaginitis (AV), VVC, or can be associated with the presence of specific sexually transmitted infections (STIs). Although clinical and microbiological features allow to categorize a variety of clinical affections and thus providing a theorical definition, in common practice, different species of pathogens, both aerobic, anaerobic and fungi usually coexist, leading to a multitude of clinical traits, that hardly can be conformed to strict models (3, 42–44).

Our results support the effectiveness of the probiotic *L. rhamnosus* CA15 (DSM 33960) strain to restore the vaginal microbiota and consequently, an effective normalization of the physiological pH, accompanied by remission or attenuation of clinical signs and symptoms as well as an improvement of quality of life in women with vaginal dysbiosis. Moreover, our study confirms a high heterogeneity in the intensity of symptoms among women with vaginal dysbiosis. Indeed, the interaction of *Candida* species with vaginal bacteria could influence the development and severity of symptoms. According to our results, change in the characteristics of the discharge (colour, consistency, amount) was most frequently reported, leading to vaginal discomfort. Moreover, patients with a predominant anaerobic microbiota reported a genital fishy odour more often than those with a predominance of aerobic bacteria; on the other hand, when *Candida* spp is significantly increased, genital itching occurs. That reason could explain the heterogeneity of clinical findings recorded in our study at the baseline. In the case of vaginal pH, for example, it is known that it is usually increased in bacterial vaginitis and therefore it often has been used as an index for altered microbiota in screening programs (45, 46). On the other hand, in patients with a microbiota dominated by *Candida*, a normal vaginal pH could be found and, even more, some authors have sustained that *Candida* infection occurred more frequently in women with vaginal pH of 4.0 or less (47). In practice, however, we can encounter a predominance of *Candida* also in women with increased vaginal pH (45), which confirms that mixed infections of *Candida* with BV, AV or both are frequent.

Our study demonstrated that the probiotic *L. rhamnosus* CA15 (DSM 33960) strain can be taken on a daily basis for 10 days without any side effects. Moreover, the treatment resulted in the restoration of the homeostasis of the vaginal microbiota and was demonstrated by the significant increase of lactobacilli combined to the reduction of pathogens. The presence of a balanced bacterial biota 30 days after the stop of the treatment indicates a lasting effect over time. According to previously reported data, probiotic strains, orally administrated, can restore the vaginal bacterial balance based on the hypothesis of bacterial translocation from the colon to the vagina (46, 47).

Actually, there has been limited information about the impact of vulvovaginal symptoms on the quality of life and the effects of probiotic treatment in women with vaginal dysbiosis. A large-scale study involving 512 women with vulvovaginal candidiasis reported a substantial negative impact on quality of life (48, 49). More recently, a randomized placebo-controlled study demonstrated the key role of specific lactobacilli strains against VVC in pregnant women and their efficacy in reducing vulvovaginal symptoms and improving emotional and social distress (50).

To our knowledge, our study is one of the first randomized-controlled trials to analyze such essential aspects in fertile age women.

Our results support the clinical evidence that vaginal dysbiosis has a negative impact on the quality of life. Women with vaginal dysbiosis might have their QoL affected in different ways, depending not only on the severity of the symptoms but also on their activities and main areas of interest (51).

Concerning the single category of QoL, the lowest mean scores at baseline were found in the **“**physical health” and **“**environmental quality of life” domains. It is reasonable to assume that vaginal symptoms lead the woman to adjust their daily activities to minimize the occurrence of discomfort. Although in our study no statistically significant differences were observed in the domain of psychological health, maybe for the shortness of the observational period, it is important to consider that, persistent symptoms could have an impact also on psychological health (52).

Questions number 20 and 21 of the WHOQOL-BREF questionnaire have evaluated social impacts attributed to vulvovaginal symptoms such as personal interactions with others and satisfaction with sexual life. Sexuality is an essential aspect of a woman's life and the quality of sexual life inevitably affects the general well-being of people (53, 54). Female sexual dysfunctions (FSD) comprise a wide range of disorders typically characterized by a clinically significant disturbance in the ability to respond sexual stimuli. They can be multidimensional and are often coexisting (55). The presence of vulvovaginal symptoms associated with vaginal dysbiosis may cause FSD because of embarrassment, and psychological distress that in turn, could impact on relationship with the partner. Of course, the psychological dimension can modify genital arousal and it is not uncommon to observe a decrease in sexual desire and arousal in women suffering from coital pain (56).

In conclusion, the administration of the *L. rhamnosus* CA15 (DSM 33960) probiotic strain can be considered an effective and safe strategy for vaginal dysbiosis. Further studies with a larger number of patients and related clinical outcomes will be mandatory to confirm these findings and to identify strategic areas for future research. Furthermore, the lack of evidence-based clinical guidelines for the management of different aspects of dysbiosis as well as the promotion of antibiotic stewardship need to be addressed. New insights into the differential diagnosis and management of abnormal vaginal microbiota will be a major challenge for future research. Finally, in view of appropriate and effective treatment choice, it becomes necessary to know how several factors influence the woman's self-perception of their problem; this will maximize the probability of successful treatment outcome.

## Data Availability

The raw data supporting the conclusions of this article will be made available by the authors, without undue reservation.
